# Transabdominal Laparoscopic Ureteroureterostomy With the Intraoperative Retrograde Ureteroscopy-Assisted Technique for Multiple Ureteral Polyps: A Single-Center 10 Years Experiences

**DOI:** 10.3389/fsurg.2022.814290

**Published:** 2022-02-25

**Authors:** Weiping Xia, Xiang Chen, Bingsheng Li, Hequn Chen, Zewu Zhu, Yao He, Yu Gan, Bo Zhang, Kangning Wang, Yang Li, Zexiang Jiang, Jin Long, Zhi Chen

**Affiliations:** ^1^Department of Urology, Xiangya Hospital, Central South University, Changsha, China; ^2^National Clinical Research Center for Geriatric Disorders, Xiangya Hospital, Central South University, Changsha, China; ^3^Department of Urology, The Second Affiliated Hospital, Guizhou Medical University, Kaili, China

**Keywords:** ureteral polyps, multiple, hydronephrosis, transabdominal laparoscopic ureteroureterostomy, retrograde ureteroscopy, outcome

## Abstract

**Background:**

The purpose of this study was to report our experience in treating multiple ureteral polyps with transabdominal laparoscopic ureteroureterostomy (LAP-UU) with intraoperative retrograde ureteroscopy (RU)-assisted technique.

**Methods:**

The data of 32 patients who underwent transabdominal LAP-UU with the intraoperative RU-assisted technique due to multiple ureteral polyps between January 2011 and March 2021 were reviewed at our institute. After administration of anesthesia, patients were placed in a passive position and underwent a three-port transabdominal laparoscopy with RU. Detailed data were reviewed, such as demographic characteristics, intraoperative outcomes, postoperative data, complications, and pathology reports.

**Results:**

Thirty-two patients were diagnosed with multiple ureteral polyps underwent this surgery method at our institution. The mean duration of symptoms at the time of diagnosis was approximately 7.1 months. The mean age of patients was 42.4 years, with men accounting for 68.8% (22/32), lesion of left for 56.3% (18/32), and the upper ureter for 62.5% (20/32). Furthermore, the median length of the polyps was 3.6 cm, the mean operative time was 174.6 min, and the estimated blood loss (EBL) was about 86.8 ml. The mean time to begin a liquid diet and to be out of bed were 1.7 and 2.3 days, respectively. The average length of hospital stay was 6.3 days. The ureteral stent was removed by cystoscope 2–3 months after surgery. Follow-up duration ranged from 3 to 112 months and none of the patients required another surgery for recurrence.

**Conclusion:**

Transabdominal LAP-UU combined with the intraoperative RU-assisted technique is an effective, safe, and reliable surgical option for patients with multiple ureteral polyps. Further long-term follow-up is recommended.

## Introduction

Ureteral polyps are rare benign tumors originating from the ureteral epithelium or non-epithelium that can cause intrinsic ureteral obstruction and related symptoms. The precise etiology of multiple ureteral polyps is not fully understood by us, and the pathological types of ureteral polyps can be divided into inflammatory polyps and fibroepithelial polyps (FEPs). The main symptoms of multiple ureteral polyps are ipsilateral flank pain and macro/micro-hematuria (1, 2). Multiple ureteral polyps always presented as many branches and stalks, and most polyps look like the head of an anemone or octopus (3). However, some multiple ureteral polyps are asymptomatic or mimic other ureteral pathologies, thus, remaining undetected or inaccurately diagnosed, and may eventually lead to renal failure throughout their lives in some patients.

With the advent of the minimally invasive era, many minimally invasive methods, such as ureteroscopy and robotic and laparoscopic surgery, have gradually replaced most open surgeries in urology. Multiple reports have described the use of ureteroscopy with holmium laser or thulium laser to treat ureteral polyps (4–7). However, this technique is mainly used to treat single or small polyps and is not effective for multiple large polyp cases, and the ureter might be injured intraoperatively due to laser heat release, and limited operating field may result in postoperative ureterostenosis or incomplete resection of the polyps. In addition, the transabdominal laparoscopic ureteroureterostomy (LAP-UU) technique has also been reported as a minimally invasive surgical approach for the treatment of ureteral obstruction resulting from ureteral polyps (8, 9). In this procedure, the surgeon often has to blindly open the ureter intraoperatively, which can lead to the ureter opening in the wrong position, a long segment of the ureter being removed, or incomplete resection of polyps. The traditional view blindly performs a completely dismembered ureter before exposure of the polyp base, which may lead to increased anastomotic tension at the end of the ureter after resection or even failure to anastomose. Hence, it is really important to find the correct position of the diseased segment of the ureter during the operation. Transabdominal LAP-UU with intraoperative retrograde ureteroscopy (RU) has emerged over time. Although some successful cases of transabdominal LAP-UU with intraoperative RU-assisted technique management have been reported in ureteral injury or inverted-Y ureteral duplication and achieved good results (10, 11), few studies have described the use of this technique in the management of multiple ureteral polyps to date.

In this article, we describe our surgical technique in detail and present our experience with this surgical technique for the treatment of ureteral obstruction caused by multiple ureteral polyps. To our knowledge, this is the first study to report transabdominal LAP-UU with the intraoperative RU-assisted technique applied for multiple ureteral polyps.

## Patients and Methods

### Patients

From January 2011 to March 2021, 32 patients were diagnosed with ureterostenosis preoperatively and were pathologically diagnosed with multiple ureteral FEPs or inflammatory polyps postoperatively underwent transabdominal LAP-UU with intraoperative RU assisted by three experienced surgeons at our institution. We retrospectively reviewed the patients' medical data and collected their clinicopathological data. All patients underwent preoperative CT, urinary ultrasonography (US), and intravenous urography (IVU) to evaluate the location and length of obstruction. Imaging findings always show that the multiple ureteral polyps are mostly uniform density and smooth edges and do not invade the adjacent tissues or organs (Supplementary Figure 1). The patient' clinical data are collected and summarized is shown in Table 1.

**Table 1:** Demographic data.

	**Total**
Gender (M/F)	22/10
Mean age (years)	42.4 ± 16.5
BMI (mean ± SD)	22.8 ± 3.4
Side of lesion (L/R)	18/14
Location of lesion (upper/middle/lower ureter)	20/5/7
Previous ureter surgery, *n* (%)	1 (3.1)
Disease course (mean ± SD, month)	7.1 ± 11.4
Degree of preoperative hydronephrosis (mild/moderate/severe)	11/11/10

*M, male; F, female; BMI, body mass index; L, left; R, right; SD, standard deviation*.

The ureteral stent was removed by cystoscope, 2–3 months after surgery. At follow-up, all patients underwent retrograde pyelography (RGP) 2 weeks after the ureteral stent was removed, and US checks every 3 months to observe for hydronephrosis changes during the first year in the outpatient department and every 6 months thereafter. In addition, other means of examination, such as IVU and CT, were performed whenever necessary, during follow-up. Surgical success was evaluated based on the improvement of hydronephrosis or the absence of further aggravation and the alleviation of symptoms.

All procedures performed in this study were approved by the Ethics Committee of Xiangya Hospital, Central South University (no.: 202103051), and informed consent was obtained from all the patients.

### Operative Technique

**Figure 1:**
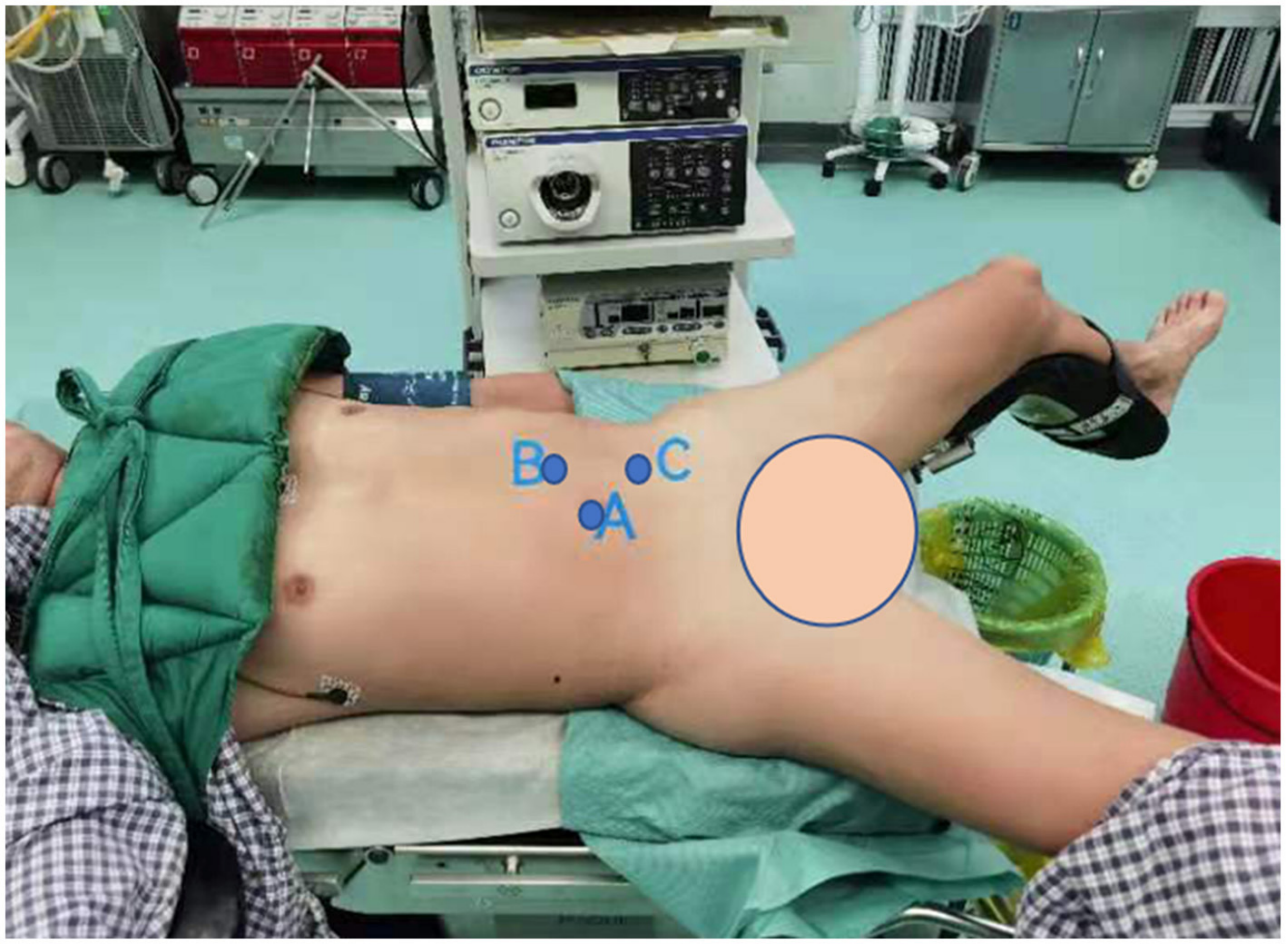
Port position setup for the left lateral transabdominal laparoscopic approach. **(A)** A 12 mm trocar at the umbilicus; **(B)** the lateral margin of the rectus, 3–4 cm above the umbilicus; **(C)** midpoint between the navel and the anterior superior iliac spine.

**Figure 2:**
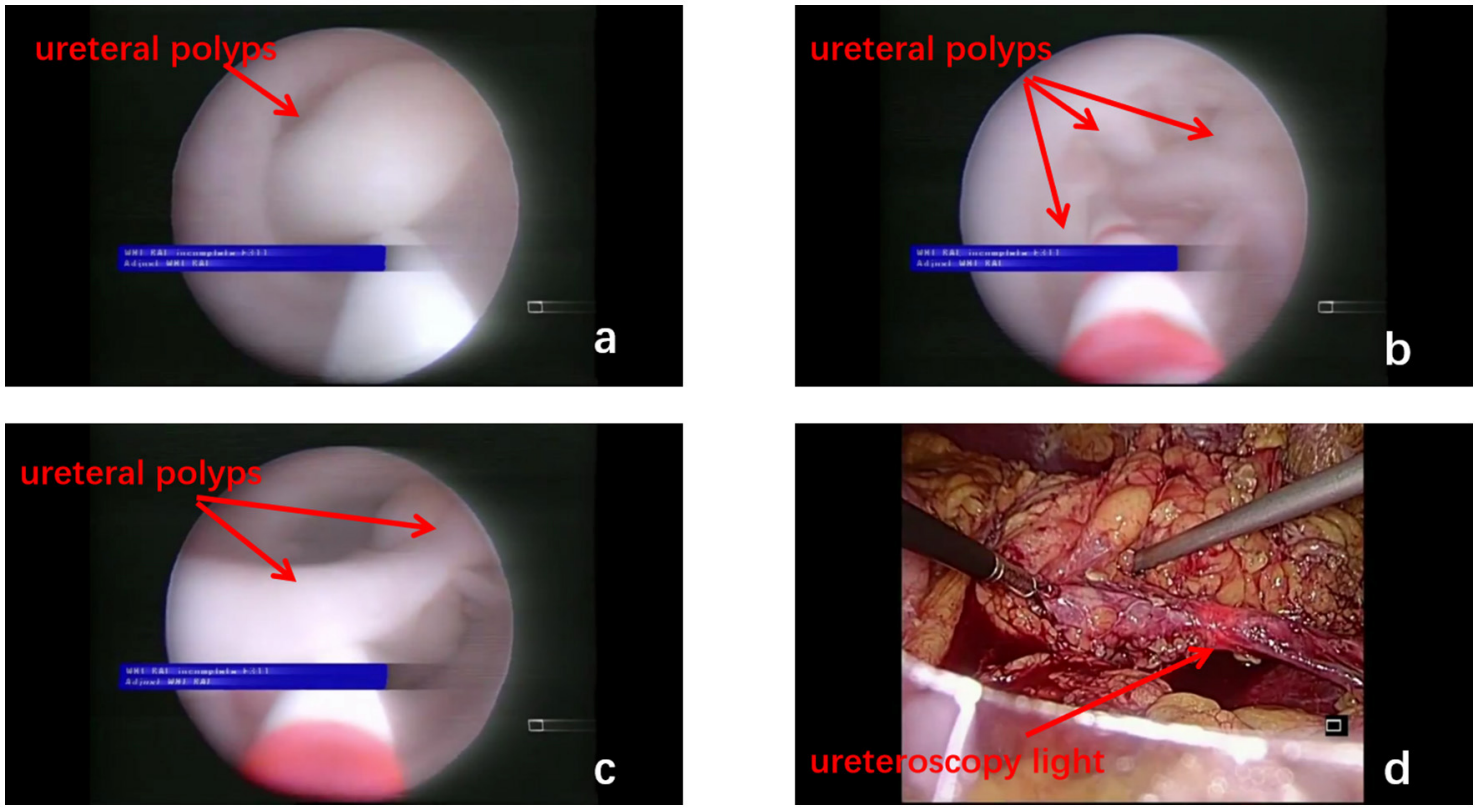
Multiple polyps in the ureteral lumen are seen under the ureteroscope (**a–c**, red arrow). The ureteroscopy light source can be clearly seen under the laparoscope (**d**, red arrow).

**Figure 3:**
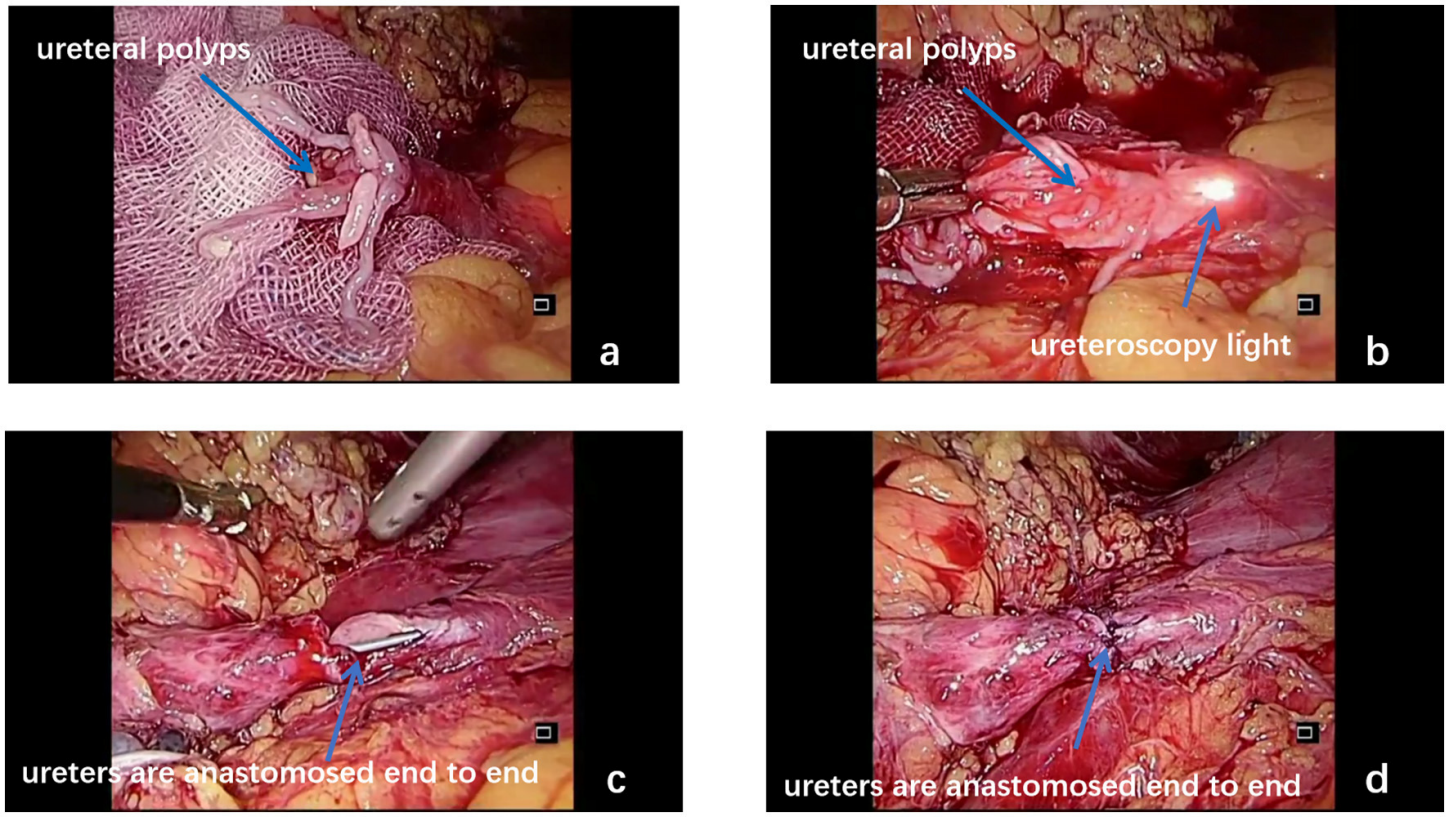
Ureteral polyps bulged out from the upper end of the ureter (**a**, blue arrow). Under the combination of the laparoscope and ureteroscope, the diseased ureteral segment is dissected longitudinally and then completely removed (**b**, blue arrow). The end-to-side anastomosis of the ureter is performed under laparoscopic guidance (**c,d**, blue arrow).

After endotracheal intubation following general anesthesia, patients were then passively placed in a position, which is a hyper extensive lithotomy position with the body in a 45 recumbent position on the surgical side up. The abdomen and perineum were disinfected together, and an arcuate incision of ~2 cm was made at the umbilicus (Figure 1, point A), followed by the insertion of a Veress needle and inflation of the umbilicus with carbon dioxide to maintain the intra-abdominal pressure at 12–14 mmHg, as described by Tang et al. (12). A 12 mm trocar for the laparoscope was then placed through the incision. Under the guidance of the laparoscope, a second trocar was inserted into the lateral margin of the rectus, 3–4 cm above the umbilicus (Figure 1, point B). A third trocar was placed at the midpoint between the navel and the anterior superior iliac spine as the working ports (Figure 1, point C). Once a minimally invasive working channel was established, we carefully dissected the ureter *via* laparoscopically and introduced a rigid ureteroscope for guidance until it was pushed to the site of polyps (Figures 2a–c). When the laparoscopic light source was dimmed, the signal from the ureteroscopy light source could be clearly seen under the laparoscope (Figure 2d). Hence, the surgeon could accurately distinguish the location of the ureteral polyps under the dual vision of the rigid ureteroscope and laparoscope. After the diseased segment of the ureter was completely freed, we opened the diseased ureter segment longitudinally with the base of the polyp stalk was fully excised at the ureteroscopy light source (Figures 3a,b). The healthy ureter was then adequately dissociated to facilitate tension-free ureteroureteral anastomosis under direct laparoscopic vision. The end-to-end anastomosis of the ureter was sutured with four to eight interrupted sutures by using resorbable 5/0 or 4/0 polyglactin material (Figures 3c,d). A 5F or 6F Double-J stent was placed during the ureteral suture procedure using a cystoscope. Confirming the presence of the Double-J stent in the bladder was achieved by visualization of the “J” shape on ureteroscopy, and a foley urethral catheter was inserted to aid in bladder emptying. Finally, one or two abdominal drainage tubes were inserted after suturing, and complete hemostasis was achieved.

The drain was removed when the volume of fluid drained was <50 ml per day. The Foley urethral catheter was removed 10–14 days postoperatively, and the ureteral stent was removed on postoperative week 12. Each resected specimen was subjected to a pathological examination to classify the type of the polyps.

### Statistical Analysis

A *t*-test was applied to analyze serum creatinine levels. The results were expressed as the number of participants (n) or percentages (%) and mean ± SD. Two-sided *p* < 0.05 was considered significant. The Statistical Package for the Social Sciences 24.0 (SPSS, Chicago, IL, USA) was used to analyze the data.

## Results

In the period of 10 years, 32 patients (male: female = 22:10) were successfully operated without conversion to open surgery for multiple ureteral polyps in our hospital. Regarding imaging examinations, all patients underwent CT scan, US, and IVU before the operation and 2 patients were diagnosed by cystoscopy for the polyps prolapse into the bladder. These patients' demographic characteristics are summarized in Table 1. The average time between the onset of symptoms and admission to the hospital was about 7.1 ± 11.4 months. Flank pain and/or hematuria are the most common presenting symptoms and some asymptomatic patients, who were found to have hydronephrosis after an incidental physical examination, were further diagnosed with multiple ureteral polyps. Multiple ureteral polyps were most found on the left (56.3%, 18/32) than on the right (43.7%, 14/32) and were more commonly located in the upper ureter (62.5%, 20/32) than in the middle ureter (15.6%, 5/32) or lower ureter (21.9%, 7/32) in patients, respectively. The mean age was 42.4 years (range 9–71 years) and these patients had hydronephrosis of different extents before surgery (mild:moderate:severe = 11:11:10). Only one patient had a history of ureteral lithotripsy with Double-J implantation previously.

**Figure 4:**
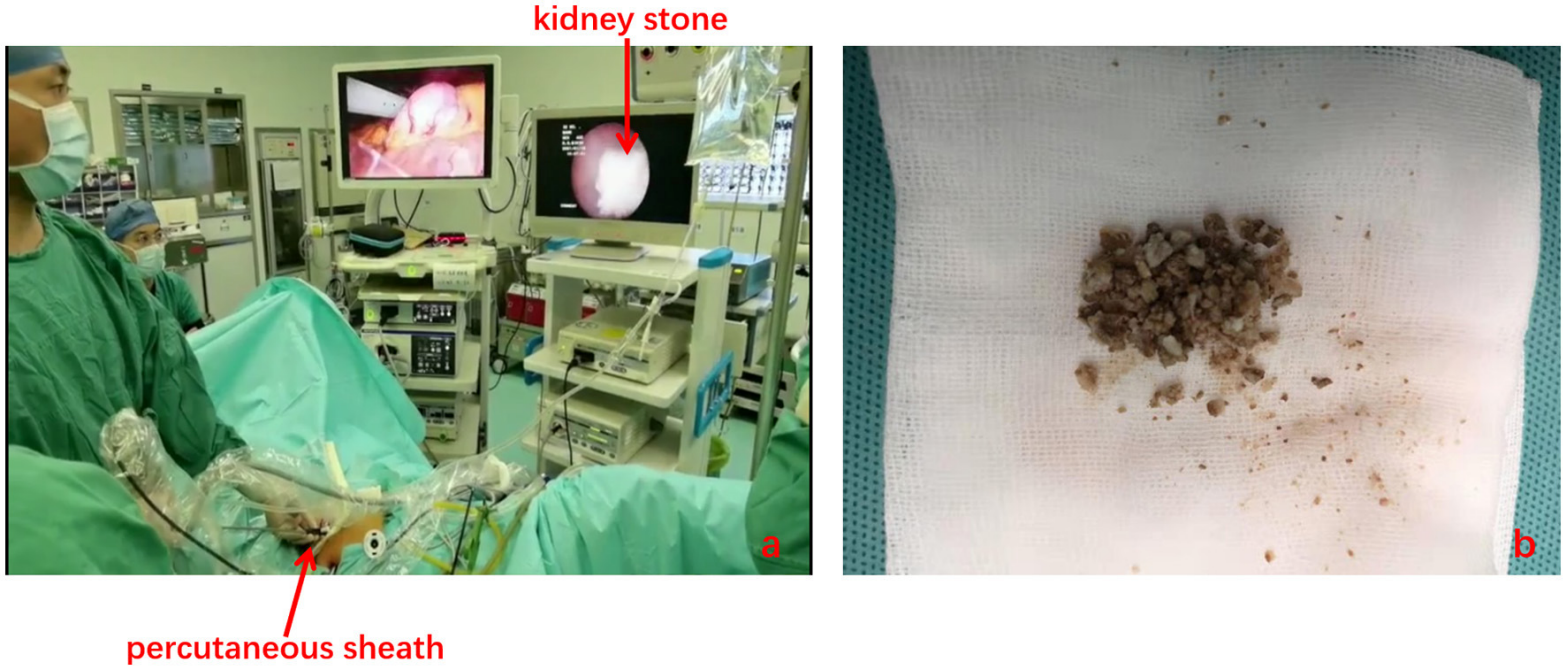
Lithotripsy is performed with the combination of the laparoscope and ureteroscope **(a)**. Kidney stone sample **(b)**.

Table 2 summarizes the intraoperative data. The mean operative time was 174.6 min, the estimated blood loss (EBL) related to the surgery was 86.8 ml, and the average length of the polyps was 3.6 cm. Most of the multiple ureteral polyps usually have multiple branches that look like anemone or octopus heads (Supplementary Figure 2). Intraoperative blood transfusion was not required in any case. In addition, 3 patients required lithotripsy intraoperative simultaneously for kidney stone, one of the patients who underwent lithotripsy intraoperative and kidney stone sample is shown in Figures 4a,b. As showed in Table 3, the average time to begin a liquid diet and to ambulation is 1.7 and 2.3 days, respectively. All patients were discharged after an average of 6.3 days. In terms of the recovery of kidney function, the mean preoperative serum creatinine was 103.8 ± 76.7 μmol/L, serum creatinine values before discharge and 3 months after discharge were 106.8 ± 87.4 μmol/L and 91.4 ± 26.5 μmol/L, respectively. There were no significant differences in the serum creatinine preoperatively, before discharge or 3 months after discharge. Postoperative pathological results showed the multiple inflammatory polyps in 26 patients and multiple FEPs in 6 patients. Five (15.6%, 5/32) patients have experienced complications after surgery, i.e., postoperative fever (6.25%, 2/32), postoperative bleeding (3.13%, 1/32), and ureteral anastomosis leaks urine (6.25%, 2/32). In two of the patients, the fever abated, and no septic shock occurred after symptomatic support therapy and regular anti-infection. One patient with postoperative bleeding was improved after conservative bedridden treatment. The other two patients suffered from urine leakage from the site of ureteral anastomosis, which prolonged their length of hospital stay. After 3 months of follow-up, the degree of hydronephrosis was relieved to some extent (no: mild: moderate: severe = 3:13:9:7). Two patients presented with urgency and frequency for the Double-J tube ectopic.

**Table 2:** Intraoperative data.

	**Total**
Operative time (mean ± SD, minute)	174.6 ± 66.6
EBL (mean ± SD, ml)	86.8 ± 53.7
Intraoperative lithotripsy (%)	3 (9.4)
Intraoperative blood transfusion (%)	0 (0)
Length of the polyps (range, cm)	3.6 (1.4–8.7)

*EBL, estimated blood loss*.

**Table 3:** Postoperative data.

	**Total**
Preoperative serum creatinine (mean ± SD, umol/l)	103.8 ± 76.7
Serum creatinine value before discharged (mean ± SD, umol/l)	106.8 ± 87.4
Serum creatinine 3 months after discharge (mean ± SD, umol/l)	91.4 ± 26.5
Time to liquid diet (mean ± SD, day)	1.7 ± 0.8
Time to out of bed (mean ± SD, day)	2.3 ± 0.8
Length of hospitalization (mean ± SD, day)	6.3 ± 2.8
Postoperative pathological results (inflammatory polyps/FEP)	26/6
Degree of hydronephrosis 3 months after operative (no/mild/moderate/severe)	(3:13:9:7)

*FEP, fibroepithelial polyps*.

The mean follow-up time was 35.8 months (range 3–112 months). It was reported that only short-term follow-up (<6.0 months) was performed in four patients. All patients visited our hospital for a US examination and RGP within 3 months. In the long-term follow-up, 13 patients were mainly followed up by telephone, and the remaining patients were regularly treated in our hospital for reexamination. Abdominal pain or gross hematuria was significantly relieved in these patients, and no recurrence of polyps was found based on the postoperative examination. During the average follow-up period, hydronephrosis was significantly decreased. Four patients with severe preoperative hydronephrosis developed renal atrophy after surgery. Moreover, two patients underwent ureteral lithotripsy for the ureteral stone formation. None of the patients required another surgery for recurrence.

## Discussion

Ureteral obstruction can be caused by extrinsic or intrinsic factors, such as a retroperitoneal tumor, ureteral endometriosis, ureteral polyps, or ureteral carcinoma (2, 13–15). Although rare, multiple ureteral polyps are the most common intrinsic causes of ureteral obstruction. However, very few case series and reports exist in the literature, on the management of ureteral polyps (16, 17). Moreover, there is almost no literature to share the experience of transabdominal LAP-UU with intraoperative RU-assisted technique that manages multiple ureteral polyps to date. Therefore, we demonstrated the presentation, diagnosis, surgical outcomes, and long-term follow-up outcomes of this technique in detail. We believe that this study comprised the largest number of patients for whom this technique was used to manage multiple ureteral polyps, with the longest follow-up duration.

The etiology of multiple ureteral polyps is still unknown to us. Some researchers believe that infection, allergic factors, chronic irritation, hormonal disorders, and congenital developmental defects may be possible etiological factors that cause polyps (9, 18). In our study, urinary tract infection was found in 16 patients on routine urinalysis; one patient had a previous history of ureteral lithotripsy with Double-J implantation. According to the position and size of the polyp, some patients remain asymptomatic while others present with flank pain, hematuria, urgency, and frequency. Hong et al. reported that approximately 58% of cases occurred in men and 75% of FEPs were reported on the left side (9). We also found a similar conclusion consistent with Hong et al. that reported and found that men accounted for 68.8% of the patients and 62.5% of lesions occurred in the upper ureter and left-side lesion prevalence for 56.3% in our study. The average length of the polyps was 3.6 cm, which is similar to the median size of the polyps reported in the literature (19).

He et al. reported that preoperative diagnosis of ureteral polyps is relatively difficult because the clinical features and image examinations lack specificity (20). Li et al. showed that the sensitivity of diagnosis of ureteral polyps was 49% by using US, MRI, and RGP (21). However, radiographic diagnosis is very important for surgical management, and the preoperative diagnosis of multiple ureteral polyps was mainly based on US, IVP, and CT in our study. Ureteral polyps always present as continuous filling defects with smooth edges on IVU and RGP, rather than the “moth-eaten” appearance of urothelial carcinoma (22). Notably, our diagnosis positive rate of multiple ureteral polyps is higher and surgeons often have their own preferences for each exam technique. We believe that an experienced imaging physician and clinic surgeon can easily diagnose multiple ureteral polyps in more than half of the patients. Simultaneously, we can perform a rapid intraoperative pathological diagnosis with the help of intraoperative RU. Therefore, we do recommend US, IVP, and CT for preoperative diagnosis.

Although, several studies have reported that ureteroscopy is helpful in treating ureteral polyps and has achieved good results. Sheng et al. demonstrated that ureteroscopy combined with thulium laser is an effective means of treating FEPs (6). Childs et al. reported the endoscopic technique management in 22 patients with FEPs (5). However, there are some disadvantages as seen in our study, where ureteral polyps were multiple and the body of polyps usually occupied the entire lumen of the ureter such that the stalk of the polyps could not be reached by the ureteroscope. Furthermore, the resection should not be too deep or wide. Moreover, poor surgical vision, the limited workspace can lead to incomplete resection or ureteral perforation (23), which might cause polyp recurrence or ureteral stricture after long-term follow-up. With the widespread application of laparoscopic techniques in urological surgeries in the past few years, its advantages are widely known. Under the broad working space and clearly surgical vision, transabdominal LAP-UU combined with intraoperative RU-assisted technique could find the exact position of polyps and simultaneously laparoscopic magnification allows the surgeon to adequate dissection of the ureter avoid unnecessary injuries and decreases the anastomotic tension. This procedure can also control the stalk of FEPs in a timely manner, thus ensuring complete resection and preventing recurrence. In addition, some patients with ureteral polyps were always accompanied with urolithiasis, 3 patients were treated with lithotripsy intraoperative simultaneously and achieved successful outcomes by using this technique in our study. After that, we can remove the stone and polyps from the trocar.

To our knowledge, transabdominal LAP-UU combined with the intraoperative RU-assisted technique is a specialized surgical technique that has not been previously applied in the management of multiple ureteral polyps; it is a technically challenging and tests the surgeon's proficiency in intracorporeal suturing and knotting, in end-to-end anastomosis of ureters. We first demonstrated the largest single-institution experience by using this technique in multiple ureteral polyps. During the long-term follow-up, the prognosis seems to be well. All patients were relieved of flank pain, hematuria, and other symptoms to some extent, and no polyp recurrence or ureteral stricture occurred within 3 months after surgery. The four patients with severe preoperative hydronephrosis developed renal atrophy after long-term follow-up. Moreover, two patients underwent ureteral lithotripsy for ureteral stone formation. We do recommend close monitoring of all patients by using IVU or US and CT for at least 1 year after surgery to exclude recurrence and ureteral stricture. Hence, transabdominal LAP-UU with RU is a safe and minimally invasive surgical technique for multiple ureteral polyps.

This study also had some limitations. First, this retrospective study included a small number of patients in a single center, despite the relatively long follow-up duration. Therefore, larger sample sizes are needed in further studies. Second, two camera systems are needed, such as ureteroscopy and laparoscopy, which may increase the financial burden on the patient. Third, the complex intraoperative suturing technique may limit its widespread application, especially in primary hospital urologists. Finally, there are few cases of multiple ureteral polyps in our center, and all of these patients use transabdominal LAP-UU with RU-assisted technique. Thus, a comparison study with an operation without RU may require validation of this technique in multiple centers in the future.

## Conclusions

Transabdominal LAP-UU combined with intraoperative RU-assisted technique can be successfully performed in patients with multiple ureteral polyps and achieved good outcomes. This technique also offers all the benefits of minimally ureteroscopy and laparoscopic surgery, such as fast recovery, clear visualization of the surgical field, and broad working space. In conclusion, this technique is an effective, safe, and reliable surgical option. Further long-term follow-up is recommended to monitor the recurrence of multiple ureteral polyps and other surgical complications to verify this technique.

## Data Availability Statement

The original contributions presented in the study are included in the article/Supplementary Material, further inquiries can be directed to the corresponding author/s.

## Ethics Statement

The studies involving human participants were reviewed and approved by the Ethics Committee of the Xiangya Hospital, Central South University (No: 202103051). Written informed consent to participate in this study was provided by the participants' legal guardian/next of kin.

## Author Contributions

WX, BL, HC, ZC, and XC: conception and design. ZC, XC, and JL: administrative support. WX, ZZ, ZJ, and YL: provision of study materials or patients. WX, YH, and YG: collection and assembly of data. WX, BZ, and KW: data analysis and interpretation. All authors: manuscript writing and final approval of manuscript.

## Funding

This work was supported by grants from the Natural Science Foundation of Hunan Province (No. 2020JJ4870), the National Natural Science Foundation of China (No. 81770693), and the Science and Technology Joint Fund project in Qiandongnan state [No. J (2020)037].

## Conflict of Interest

The authors declare that the research was conducted in the absence of any commercial or financial relationships that could be construed as a potential conflict of interest.

## Publisher's Note

All claims expressed in this article are solely those of the authors and do not necessarily represent those of their affiliated organizations, or those of the publisher, the editors and the reviewers. Any product that may be evaluated in this article, or claim that may be made by its manufacturer, is not guaranteed or endorsed by the publisher.

## Supplementary Material

Supplementary Figure 1 The red arrow shows imaging findings of multiple ureteral polyps in three patients.

Supplementary Figure 2 Postoperative specimens of multiple ureteral polyps often have multiple branches that look like the head of an anemone or octopus (**a**, black arrow. **b**, black circle).
