# Supplementary figures and images for: Transabdominal Laparoscopic Ureteroureterostomy With the Intraoperative Retrograde Ureteroscopy-Assisted Technique for Multiple Ureteral Polyps: A Single-Center 10 Years Experiences
Source: Front Surg. 2022 Feb 25;9:814290. doi: 10.3389/fsurg.2022.814290 (PMC8913589; doi:10.3389/fsurg.2022.814290)

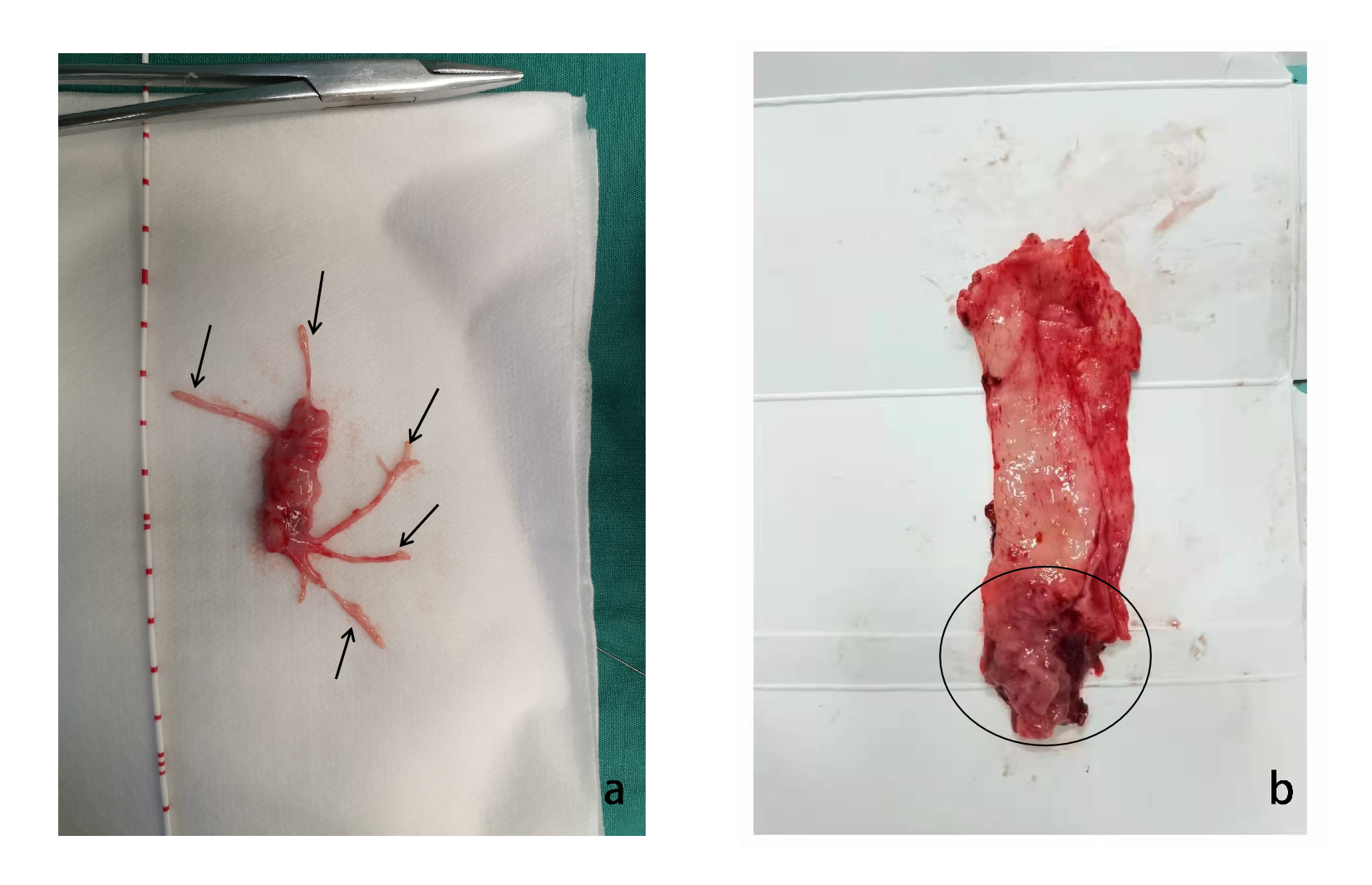

Supplement: Supplementary Figure 1 — The red arrow shows imaging findings of multiple ureteral polyps in three patients. [file Image_1.TIF]

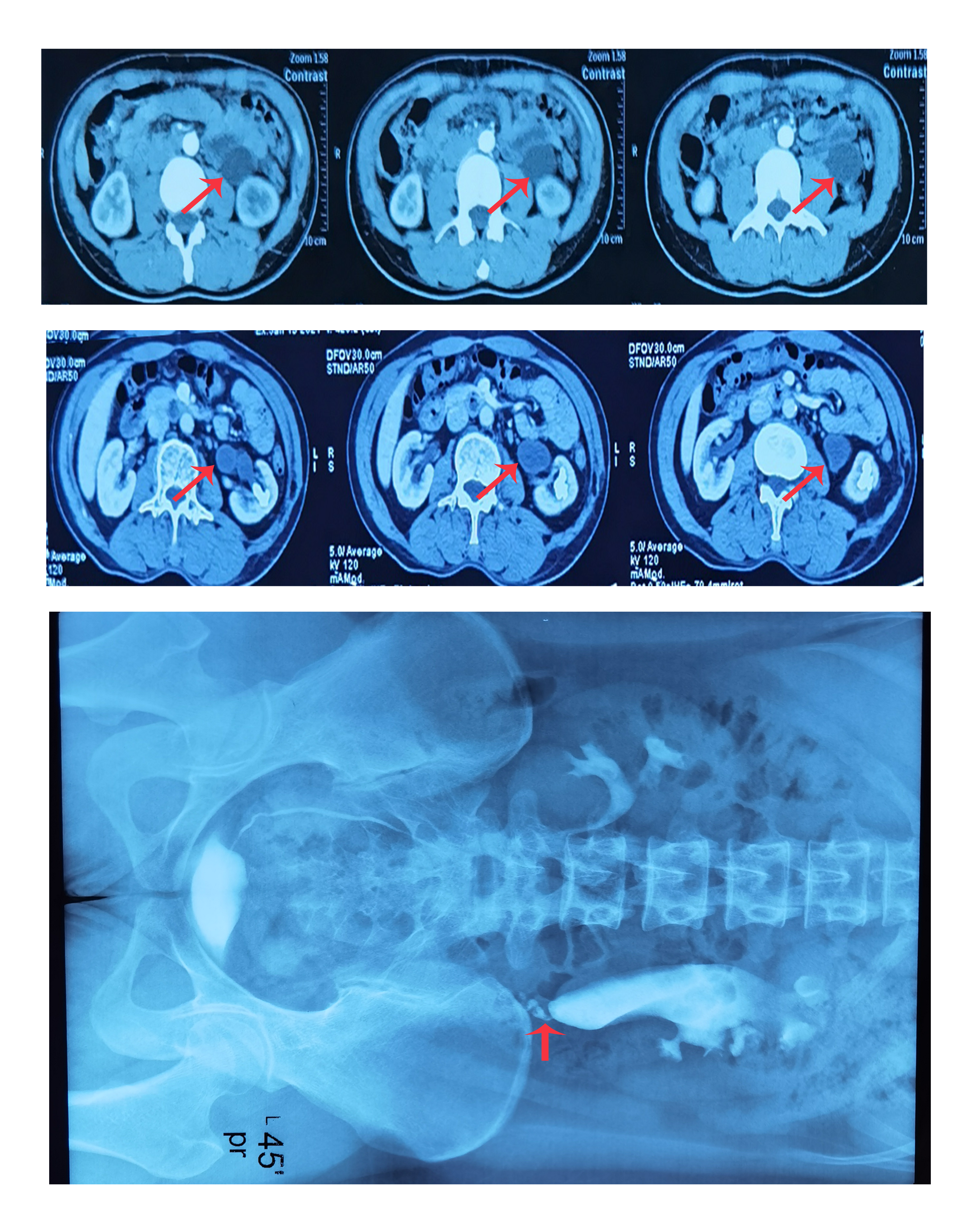

Supplement: Supplementary Figure 2 — Postoperative specimens of multiple ureteral polyps often have multiple branches that look like the head of an anemone or octopus (a, black arrow. b, black circle). [file Image_2.PNG]
